# Effectiveness of a Novel Contralaterally Controlled Neuromuscular Electrical Stimulation for Restoring Lower Limb Motor Performance and Activities of Daily Living in Stroke Survivors: A Randomized Controlled Trial

**DOI:** 10.1155/2022/5771634

**Published:** 2022-01-11

**Authors:** Ying Shen, Lan Chen, Li Zhang, Shugang Hu, Bin Su, Huaide Qiu, Xingjun Xu, Guilan Huang, Zhifei Yin, Jinyu Yang, Chuan Guo, Tong Wang

**Affiliations:** ^1^Rehabilitation Medicine Center, The First Affiliated Hospital of Nanjing Medical University, Nanjing, China; ^2^Rehabilitation Department, The Affiliated Wuxi Mental Health Center of Nanjing Medical University, Wuxi, China; ^3^Rehabilitation Department, The Affiliated Jiangning Hospital of Nanjing Medical University, Nanjing, China

## Abstract

**Background:**

Contralaterally controlled neuromuscular electrical stimulation (CCNMES) is a novel electrical stimulation treatment for stroke; however, reports on the efficacy of CCNMES on lower extremity function after stroke are scarce.

**Objective:**

To compare the effects of CCNMES versus NMES on lower extremity function and activities of daily living (ADL) in subacute stroke patients.

**Methods:**

Forty-four patients with a history of subacute stroke were randomly assigned to a CCNMES group and a NMES group (*n* = 22 per group). Twenty-one patients in each group completed the study per protocol, with one subject lost in follow-up in each group. The CCNMES group received CCNMES to the tibialis anterior (TA) and the peroneus longus and brevis muscles to induce ankle dorsiflexion motion, whereas the NMES group received NMES. The stimulus current was a biphasic waveform with a pulse duration of 200 *μ*s and a frequency of 60 Hz. Patients in both groups underwent five 15 min sessions of electrical stimulation per week for three weeks. Indicators of motor function and ADL were measured pre- and posttreatment, including the Fugl–Meyer assessment of the lower extremity (FMA-LE) and modified Barthel index (MBI). Surface electromyography (sEMG) assessments included average electromyography (aEMG), integrated electromyography (iEMG), and root mean square (RMS) of the paretic TA muscle.

**Results:**

Values for the FMA-LE, MBI, aEMG, iEMG, and RMS of the affected TA muscle were significantly increased in both groups after treatment (*p* < 0.01). Patients in the CCNMES group showed significant improvements in all the measurements compared with the NMES group after treatment. Within-group differences in all post- and pretreatment indicators were significantly greater in the CCNMES group than in the NMES group (*p* < 0.05).

**Conclusion:**

CCNMES improved motor function and ADL ability to a greater extent than the conventional NMES in subacute stroke patients.

## 1. Introduction

Stroke is one of the most frequently occurring diseases in the world, leading to permanent disability and a decline in quality of life, resulting in a heavy burden of illness [1]. There are more than 2 million new cases in China each year, 70-80% of which are unable to live independently because of disability [2]. Most stroke patients have impaired walking ability and can only walk at home, and the restoration of mobility is often one of the primary goals of rehabilitation [3].

Neuromuscular electrical stimulation (NMES) is an effective therapeutic method for facilitating the recovery of motor function in paralyzed lower limbs of stroke survivors [4]. During NMES, a low-frequency pulse current is applied over the motor nerves or muscles to mimic exercise therapy via smooth tetanic muscle contractions [5, 6]. NMES can help maintain joint range of motion (ROM), strengthen muscle, and promote motor relearning. NMES can be utilized to modulate neural activity to either restore contraction of the tibialis anterior (TA) and peroneus longus and brevis muscles or prevent an abnormal triceps surae reaction due to causes such as spasticity. NMES of the peroneal nerve provokes ankle dorsiflexion. Indeed, NMES of the peroneal nerve can be used not only to correct drop-foot but also to modulate long-term neuromuscular system function, thereby promoting the rehabilitation of lower limb function after stroke [7]. However, a major weakness of NMES is that patients cannot initiate active movement as NMES is a completely passive treatment. To better promote functional gain, NMES should be combined with subjective efforts; however, this is difficult to achieve, especially in patients with acute or severe motor dysfunction.

Contralaterally controlled neuromuscular electrical stimulation (CCNMES), an innovative NMES method, was recently used to restore ankle dorsiflexion in patients with chronic stroke [8]. A few groups have begun to investigate the impact of CCNMES on ankle dorsiflexion in hemiplegic stroke patients [9]. In one study, Knutson and colleagues used a sensor attached to patient's sock that was worn on the nonparetic foot to measure the angle of ankle activity, as well as two surface electrodes positioned, one positioned over the peroneal nerve and the other over the TA muscle, to activate ankle dorsiflexion motion. The current stimulus intensity was directly proportional to the degree of active ankle dorsiflexion of the unaffected side [8]. It was also reported that both CCNMES and NMES plus walking training could improve lower limb motor function in patients with chronic stroke [10]. CCNMES uses the joint angles or electromyographic (EMG) signal from the nonparetic side for a predefined movement to control the intensity of the electrical stimulation delivered to the paretic side to induce a similar movement [11, 12]. The major differences between CCNMES and NMES are as follows: (i) the range of motion and speed of affected limb movement are in the control of patients themselves; (ii) bilateral symmetrical joint movement [10, 12]. However, the clinical benefit of using CCNMES to restore hemiplegic lower limb function remains unclear.

Here, we applied the CCNMES that uses an electromyographic EMG signal instead of an angle sensor attached to patient's sock to trigger the stimulator. The intensity of the electrical stimulation delivered to the peroneal nerve and TA muscle was inducted by the electromyographic value instead of the degree of volitional dorsiflexion. The objective of this study was to investigate whether the application of this innovative CCNMES method could restore lower limb motor function in hemiplegic patients.

## 2. Materials and Methods

### 2.1. Design

This study was a randomized controlled trial designed to compare the clinical outcomes between CCNMES and NMES on motor function recovery after stroke. Forty-four patients were randomly assigned to two groups in a 1 : 1 ratio using a simple randomization procedure.

### 2.2. Subjects

Participants with lower-extremity hemiplegia were recruited from Wuxi Tongren Rehabilitation Hospital from April 2021 to July 2021. The criteria for inclusion were as follows: (i) diagnosis of stroke was confirmed by computed tomography (CT) and/or magnetic resonance imaging (MRI); (ii) initial, unilateral onset or previous episodes without neurological impairment between 1 and 6 months poststroke; (iii) aged 40 to 80 years; (iv) a grade of ankle dorsiflexion strength ≤3/5 (Lovett scale); (v) lower extremity Brunnstrom stage I–III; and (vi) the hemiplegic side with normal passive ankle movement. The criteria for exclusion were (i) progressive stroke with nonstable condition; (ii) cerebellar or brainstem stroke; (iii) unable to cooperate with assessment and treatment due to severe cognitive and communication impairment; (iv) complication with severe heart, lung, liver, kidney, or infectious disease; (v) the wearing of a cardiac pacemaker; and (vi) motor dysfunction due to other causes before the onset of stroke.

This study was approved by the Ethics Committee of the Affiliated Wuxi Mental Health Center of Nanjing Medical University (Wuxi Tongren Rehabilitation Hospital) (No.: WXMHCIRB2020LLky040). This trial was registered in the China Clinical Trial Registration Center (ChiCTR2100045143). All patients provided signed informed consent.

### 2.3. Electrical Stimulation Equipment

The electrical stimulator (S4, Vishee Co., Nanjing, China) delivered pulses (pulse width 200 *μ*s, biphasic asymmetrical rectangular) at 60 Hz. The CCNMES device consisted of a stimulator and a electromyographic sensor. The electromyographic sensorn the unaffected limb is used to trigger the stimulator. One recording electrode was placed below the head of the fibula over the common peroneal nerve and another at the motor point of the unaffected TA muscle [10]; a third reference electrode was placed in the middle. For the hemiplegic side, one stimulating electrode was placed below the head of the fibula over the common peroneal nerve and another at the motor point of the TA muscle, with one reference electrode in the middle [10]. The patients were not supposed to feel pain due to the administered electrical stimulation.

**Figure 1:**
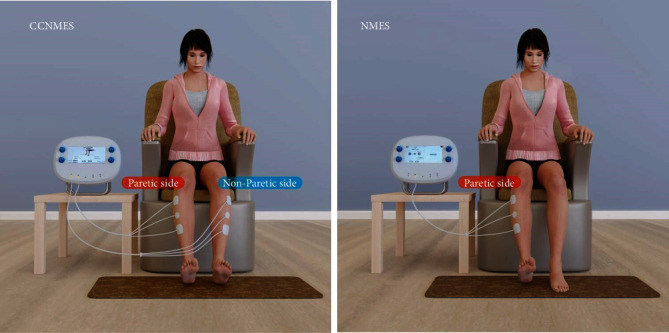
Graphical presentation of contralaterally controlled neuromuscular electrical stimulation (CCNMES) and neuromuscular electrical stimulation (NMES).

For the CCNMES treatment (Figure 1), the intensity of the electrical stimulation applied to induce dorsiflexion in the hemiplegic ankle was modulated by the EMG value of the contraction of the unaffected TA muscle [10, 12]. Before treatment, the patients were asked to dorsiflex their unaffected ankle to almost 10% ROM and hold that position. The electrical equipment then recorded the EMG value of the muscle contraction obtained by the sensor attached to the recording electrodes. Simultaneously, an operator increased the stimulating current in a stepwise fashion until the current was sufficiently strong to induce dorsiflexion of up to 10% ROM in the hemiplegic ankle. The intensity of the stimulating current was also automatically recorded by the equipment. Thus, the EMG value of the muscle contraction of the affected side corresponded to the electric current intensity in the contralateral unaffected muscle. The same procedure was then applied with patients dorsiflexing their nonparetic ankle to 50% and 100% ROM. Once these three steps had been completed, the current intensity of the hemiplegic side corresponding to the EMG value of the unaffected side could be calculated and recorded by the electrical stimulation equipment. Before the CCNMES treatment, the patients were taught how to use the equipment by their respective physiotherapists so they could perform the electrical stimulation by themselves. During the treatment, the patients had to try to dorsiflex both ankles after hearing the cueing tone. As the EMG value of the unaffected ankle dorsiflex was detected, the electrical instrument immediately stimulated the affected peroneal nerve and the motor point of the unaffected TA muscle using the proportional current intensity calculated by the instrument [12]. This resulted in both ankles being able to complete the same action almost simultaneously in the treatment. For the NMES treatment (Figure 1), the stimulation sites and parameters of the hemiplegic limb were the same as those for the CCNMES treatment.

### 2.4. Intervention

Patients in both groups underwent five 15 min sessions of electrical stimulation per week for 3 consecutive weeks. All patients received routine rehabilitation treatment. Each CCNMES pulse lasted for 15 s with 10 s breaks for a total of 36 pulses. The patients repeatedly attempted to stretch both ankles according to the sound cues until full ankle dorsiflexion was achieved for 15 s with a 10 s resting period. Each NMES pulse lasted 15 s with 10 s breaks for a total of 36 pulses.

### 2.5. Allocation and Assessment

Patients were assigned to the CCNMES or NMES groups at a 1 : 1 ratio using simple randomization. The randomization sequence was generated before the treatment, and the information was concealed in sequentially numbered, sealed, opaque envelopes. Patients selected an envelope after signing the informed consent form, following which the staff, who were blinded to the trials, informed the physiotherapist about the allocated intervention regimen. All the outcome measures were assessed pre- (T1) and posttreatment (T2; at the end of the 3-week treatment).

### 2.6. Outcome Assessment

The primary outcome measure was the Fugl–Meyer assessment of the lower extremity (FMA-LE). Secondary outcomes included the modified Barthel index (MBI) score, as well as the average electromyography (aEMG), integrated electromyography (iEMG), and root mean square (RMS) values for the affected TA muscle.

#### 2.6.1. FMA-LE

The FMA-LE was used to assess the motor function of the lower limb [13]. The maximum score for the whole FMA-LE was 34. The higher the score, the better the motor function in the paretic lower limb.

#### 2.6.2. MBI

The MBI score was used to evaluate the ability to perform activities of daily living (ADL). The maximum score for the whole MBI assessment was 100. The higher the score, the better the level of physical function [14].

#### 2.6.3. sEMG

A wireless BTS-FREEEMG 300 (BTS Bioengineering, Milan, Italy) was used to collect the surface electromyography (sEMG) signals of the paretic TA muscle, which were synchronously transmitted to a BTS EMG-Analyzer (BTS Bioengineering) [15]. Patients were asked to sit comfortably in a chair with knees flexed at 90 degrees, ankles in an anatomically neutral position. Before data collection, the skin was cleaned using alcohol and shaved if needed. A pair of silver–silver chloride electrodes (H124SG, 30 mm × 24 mm; COVIDIEN-Kendall, Waukegan, IL, USA) were placed on the belly of the TA muscle with an interelectrode distance of 2 cm while a reference electrode was placed on the affected ankle. Before the sEMG recording, patients were taught how to perform the maximum voluntary isometric contraction (MVIC) of ankle dorsiflexion until they could complete it correctly [16]. For each patient, three 3 s trails of MVIC were recorded with a 2 min rest between each trail. The sampling frequency of the sEMG signals was 1,000 Hz, and the signals were recorded for off-line analysis.

EMG signals were automatically analyzed by the BTS EMG-Analyzer with the band-pass filtered from 20 to 500 Hz. aEMG is the average of the instantaneous amplitude of the 3 s MVIC, which represents the degree of muscle activation during movement and the type and synchronization of activated motor units [17]. iEMG is defined as the total magnitude of discharge of motor units involved in muscle activities in the 3 s period and partially reflects the number of involved motor units and their discharge in that period [18]. RMS, the mean square root of the square of the amplitude of instantaneous EMG signals in a 3 s period, is determined by changes in the amplitude and reflects the recruitment of motor units [19].

#### 2.6.4. Sample Size Calculation

The sample size was calculated using G∗Power Software (version 3.1.9.4). The effect size (*f*) was calculated as 1.095 based on the FMA-LE score from a group of individuals as previously reported [20]. To achieve 90% power with an alpha error of 5%, a minimum sample size of 38 patients (19 per group) was needed to detect statistical significance for a between-group difference in FMA-LE. Assuming a 10% dropout rate, the minimum number of enrolled patients was determined to be 44 (22 per group).

## 3. Statistical Analysis

Statistical analysis was performed using SPSS23.0 (IBM Inc., Chicago, IL, USA). Enrolled patients and drop-out cases with corresponding reasons were recorded. Clinicodemographic data were summarized using descriptive statistics. Specifically, counts and percentages were applied for categorical variables. The continuous data were checked for normality using the Shapiro-Wilk test; continuous data were presented as means with standardized deviation (SD) when data conformed to normal distribution; otherwise, median and interquartile range were applied. Paired *t*-tests were used for intragroup comparisons, while independent samples *t*-tests were used for comparisons between groups. For nonnormally distributed data or data lacking homogeneity of variance, the Wilcoxon rank-sum test (Mann–Whitney *U* test) was used to compare paired and between-group samples. *p* < 0.05 was considered statistically significant.

## 4. Results

### 4.1. Participants

**Figure 2:**
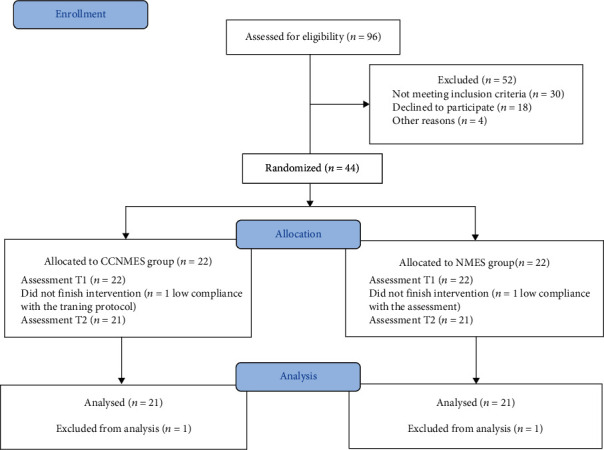
CONSORT flow diagram.

A total of 44 stroke patients were randomly divided into a CCNMES group and a NMES group (22 patients per group) using SPSS software. One patient in the CCNMES group withdrew due to low compliance with the training protocol, while one patient in the NMES group withdrew owing to their failure to complete the sEMG evaluation. The progress of patients throughout the study is shown in the CONSORT flow diagram (Figure 2). No significant differences in baseline data were found between the two groups (*p* > 0.05) (as detailed in Table 1).

### 4.2. Motor Performance and ADL

As presented in Table 2, no significant differences in FMA-LE and MBI scores were observed between the two groups at baseline (*p* = 0.269 and 0.732, respectively). Within-group differences in FMA-LE scores for both the CCNMES and NMES groups were statistically significant after treatment (*p* < 0.001); a between-group comparison indicated that the improvement in the CCNMES group was significantly better than that of the NMES group (difference = 1.71, 95% CI 0.47–2.96, *p* = 0.009). Similarly, CCNMES was associated with a significantly greater improvement in the MBI score as compared with NMES (difference = 6.10, 95% CI 0.84–11.35, *p* = 0.024), while within-group improvements were observed in both groups (*p* < 0.001) (Table 2).

**Figure 3:**
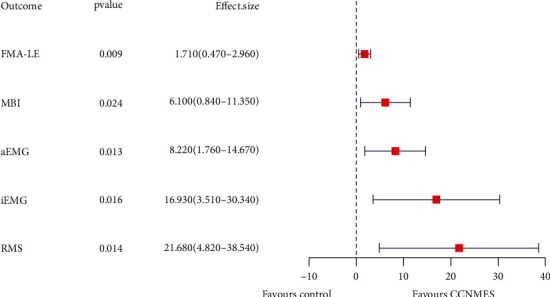
Forest plot presenting the treatment effects of CCNMES and NMES on the different parameters assessed. FMA-LE: Fugl–Meyer assessment-lower extremity; MBI: modified Barthel index; aEMG: average electromyography; iEMG: integrated electromyography; RMS: root mean square.

The forest plot (Figure 3) presenting treatment effects of CCNMES and NMES in the FMA-LE and MBI assessment also showed greater benefits for CCNMES. For the FMA-LE, the effect size was 1.710 (95% CI 0.470–2.960, *p* = 0.009) favoring CCNMES, while for MBI, the effect size was 6.100 (95% CI 0.840–11.350, *p* = 0.024), also favoring CCNMES.

### 4.3. sEMG

As depicted in Table 3, no significant differences in aEMG, iEMG, or RMS values were detected between the groups at baseline (*p* = 0.660, 0.575, and 0.473, respectively); however, after electrical stimulation, the above three sEMG values were significantly increased in both groups (*p* < 0.001). Additionally, a between-group comparison indicated that patients in the CCNMES group displayed significantly improvements in the assessed parameters compared with those of patients in the NMES group (for aEMG: difference = 8.22, 95% CI 1.76–14.67, *p* = 0.013; for iEMG: difference = 16.93, 95% CI 3.51–30.34, *p* = 0.016; for RMS: difference = 21.68, 95% CI 4.82–38.54, *p* = 0.014).

The increase in the aEMG value from baseline to posttreatment for the CCNMES group was higher than that for the NMES group (effect size = 2.67, 95% CI 1.760–14.670, *p* = 0.013). A similar pattern could be seen in the iEMG and RMS assessments (increases = 16.93, 95% CI 3.510–30.340, *p* = 0.016; and 21.68, 95% CI 4.82–38.54, *p*  = 0.014, respectively) (Figure 3).

## 5. Discussion

In our study, we compared the efficacy of CCNMES versus NMES on the lower limb function and ADL in stroke survivors. Our results indicated that although FMA-LE, MBI, and sEMG values were significantly improved in both groups after treatment, patients in the CCNMES group exhibited better recovery than those in the NMES group. This suggested that CCNMES might be more effective at promoting the recovery of lower limb function in hemiplegic patients and improvements in ADL abilities. In addition, we also recorded sEMG activity in the TA muscle. Significant increases in the aEMG, iEMG, and RMS values were observed in both groups, with patients in the CCNMES group again showing the best results. NMES is effective at improving muscle fiber recruitment [21]. The sEMG data in our study support that CCNMES was better at increasing the number and synchronization of activated motor units during TA muscle contraction.

To date, relatively few studies have investigated the efficacy of CCNMES on the recovery of lower limb function after stroke. Knutson and Chae were the first to report on the feasibility of using CCNMES at the paretic ankle to improve lower limb function in patients with chronic stroke [8]. Despite the small sample size, the results of that study indicated that CCNMES may exert positive effects on the functional recovery of lower limbs in poststroke patients. In a follow-up study, the authors randomized 26 patients with chronic stroke (≥6 months) into a CCNMES and a NMES group and found that 6 weeks of CCNMES or NMES plus walking training could improve lower limb motor function; however, neither treatment was found to be superior [10]. The reason for the different result between ours and the above-mentioned study may be due to the different disease duration of patients. In the study of Knutson et al. [10], the mean illness duration was 2.7 years in the CCNMES group, compared with 84 days in the present study. This suggests that stroke patients with a shorter disease course tend to benefit more from CCNMES than those with a longer disease course.

NMES is considered to be a promising adjuvant therapy for the rehabilitation of the motor control deficits caused by stroke. The benefits of NMES can be interpreted in terms of some possible peripheral mechanisms, including that (i) NMES can maintain and increase joint ROM, although to achieve this, the intensity of NMES must be large enough to attain maximum range with joint activity; (ii) NMES can minimize and prevent muscle atrophy [22]; and (iii) NMES can reduce spasticity by improving motor function [18, 23]. In addition, NMES may promote motor function rehabilitation after stroke through several central mechanisms. For instance, somatosensory input, in the form of peripheral nerve stimulation through electrodes applied on the skin surface, can reportedly enhance motor performance. Sensory-level somatosensory stimulation induced by NMES was found to activate large cutaneous and proprioceptive fibers, with increased somatosensory nerve input through stimulated peripheral nerve leading to improved motor control performance [17]. The peripheral stimulation of the hemiplegic limb sends neural input to the cortex of the cerebrum which induces changes in cortical plasticity in the primary somatosensory cortex, primary motor cortex, and premotor cortex [19]. Improving cortical excitability is conducive to enhancing muscle strength [24].

In this study, patients in the CCNMES group showed better motor function and ADL recovery compared with those in the NMES group. Despite the similarities between CCNMES and NMES, the former has several potential advantages over the latter. First, CCNMES is an active physical therapy method and does not require the paralyzed limbs to retain any residual motor function. In contrast, NMES, which is applied to stroke patients with acute or severe motor dysfunction, is a completely passive treatment. Passive NMES does not sufficiently fit the current theory underlying motor relearning because motor relearning requires the subjective efforts of the patient and task-oriented training [25]. During CCNMES, patients must use an active predefined movement of the nonaffected limbs to trigger the electric stimulation equipment. The intensity of the electric stimulation is directly proportional to the degree of active muscle contraction of the contralateral limb, thereby enabling the ROM and speed of the affected limb to be controlled by the patients themselves. For the hemiplegic upper extremity in stroke patients, improved motor control was reported to be easier to achieve when voluntary movements were used to trigger electric stimulation instead of conventional electric stimulation [26]. Thus, CCNMES may be better at promoting motor control ability, especially for stroke patients with subacute or severe motor dysfunction who have no or little residual motor function.

Secondly, CCNMES can potentially be used to induce synchronized presynaptic and postsynaptic activity of the affected population of anterior horn cells. It is well known that, based on this mechanism, functional electrical stimulation (FES), such as that provided by a foot-drop stimulator, can promote the recovery of motor function in hemiplegic lower limbs after stroke [18]. The key underlying the ability of electrical stimulation to induce synchronous presynaptic and postsynaptic activity lies with the simultaneous provision of electrical stimulation and patients' subjective effort [27]. Indeed, when repetitive movements controlled by the central nervous system were paired with somatosensory stimulation provided by electrical stimulation, there was a demonstrable improvement in motor function and sensory cortex activation [28]. Changes in the sensory cortex can also reflect a heightened sensitization of neurons associated with limb movement, which may behave like a long-term potentiation process [29]. The traditional and completely passive NMES would not work through this mechanism [18]. In contrast, CCNMES emphasizes intention-driven movement, thereby allowing the synchronization of motor intention and stimulated limb movement [9]. The electrical stimulation and the antidromic impulse produced by CCNMES are combined with simultaneous voluntary motor intention and efforts. This endows CCNMES with the potential for therapeutic application to artificially initiate synchronized presynaptic and postsynaptic activity in the affected anterior horn cells through activating the residual pyramidal tract.

Finally, another advantage of CCNMES over NMES is that it utilizes the “bilateral exercise” principle [12]. Bilateral exercise uses the interlimb coupling effect to take advantage of the nonaffected limb to promote motor function recovery in the affected limb [30]. To achieve this effect, both limbs of the stroke patient must be repetitively and intensively trained simultaneously [31]. A balance in interhemispheric inhibition is essential for normal voluntary movement. When a patient suffers a stroke in one hemisphere, this balance is broken, and the reduced interhemispheric inhibition from the ipsilateral to the contralateral hemisphere may result in increased inhibition from the latter to the former, thereby decreasing the excitability of the ipsilateral hemisphere [32]. Bilateral exercise poststroke may accelerate cortical neural plasticity and promote interhemispheric disinhibition, which allows better use of the spared pathways of the affected hemisphere [33]. Additionally, bilateral exercise can supplement the damaged crossed corticospinal pathways by recruiting the ipsilateral pathways from the contralesional or contralateral hemisphere [34]. Activity in the contralesional hemisphere is likely to contribute to motor function recovery after stroke *via* a small proportion (10%) of the pyramidal tract that did not decussate [35]. Cunningham et al. showed that this type of bilateral neuromuscular electrical stimulation can reduce the interhemispheric inhibition from unaffected motor cortices and maintain the ipsilesional output to the paretic limb [36]. This indicates that bilateral and unilateral electrical stimulation act through different neurophysiological mechanisms in stroke participants and that CCNMES has the potential to promote the recovery of paretic limbs through symmetric bilateral movement, which reduces inhibition between the bilateral hemispheres.

This study had several limitations. The selected patients were between 1 and 6 months poststroke, and it was uncertain whether the outcomes were affected by a natural recovery from the disease. Further studies will be needed to assess the clinical generalization of this interventional technique for stroke patients and other neurological patients with similar functional motor impairments that affect their daily activities. Another limitation of our study was the absence of a control group receiving routine rehabilitation treatment without electrical stimulation. Thus, the results observed in the NMES group could not be solely attributed to the unique effects of NMES.

## 6. Conclusions

CCNMES may involve a variety of rehabilitation principles that are crucial for promoting motor function recovery. The ability of CCNMES to promote neuroplastic changes is based on principles such as repetitive, active, and bilateral symmetric movement; neuromuscular electrical stimulation; and the activation of motor neurons, afferent fibers, and the primary motor cortex [37]. In our study, both the CCNMES and NMES interventions resulted in a marked amelioration of paretic lower extremity function and ADL ability; however, CCNMES showed better motor function and greater recovery in ADL abilities than the conventional NMES treatment. CCNMES represents an effective therapeutic method to accelerate the recovery of hemiplegic lower limbs and has the potential for use in stroke rehabilitation.

## Figures and Tables

**Table 1:** Clinicodemographic data of the study cohort at baseline.

	CCNMES group (*n* = 21)	NMES group (*n* = 21)	*t/χ* ^2^	*p* value
Age, years, mean (SD)	62.86 (12.96)	66.09 (6.38)	-1.048	0.301
Sex, *n* (% male)	18 (81.82%)	15 (68.18%)	1.091	0.296
Type of stroke				
Ischaemic, *n* (%)	15 (68.18%)	18 (81.82%)	1.091	0.296
Haemorrhagic, *n* (%)	7 (31.82%)	4 (18.18%)		
Lesion site, *n* (%left)	12 (54.55%)	10 (45.45%)	0.364	0.546
Time since stroke onset (days), mean (SD)	84.00 (39.60)	73.45 (33.15)	0.985	0.344

CCNMES: contralaterally controlled functional electrical stimulation; NMES: neuromuscular electrical stimulation; SD: standard deviation.

**Table 2:** Comparison of motor performance and ADL in two groups and at before and after treatment.

	CCNMES group Mean (SD)	NMES group Mean (SD)	*Z*	*p* value	Difference between groups (95% CI)
FMA-LE					
Before treatment	8.81 (2.52)	7.95 (2.40)	−1.106	0.269	0.86 [−0.68–2.39]
After treatment	14.48 (2.96)^a^	11.90 (3.35)^a^	−2.362	0.018	2.57 [0.60–4.54]
Difference	5.67 (2.13)	3.95 (1.87)	−2.267	0.009	1.71 [0.47–2.96]^b^
MBI					
Before treatment	39.43 (10.53)	37.05 (6.11)	−0.343	0.732	2.38 [−2.99–7.75]
After treatment	56.62 (11.96)^a^	48.14 (9.86)^a^	−2.222	0.026	8.48 [1.63–15.32]
Difference	17.19 (9.73)	11.10 (6.79)	−2.261	0.024	6.10 [0.84–11.35]^b^

SD: standard deviation; CI: confidence interval; ADL: activities of daily living; FMA-LE: Fugl–Meyer assessment-lower extremity; MBI: modified Barthel Index. ^a^*p* < 0.001, differences before and after treatment; ^b^*p* < 0.05, differences between groups.

**Table 3:** Comparison of sEMG-related indices between the two groups and between before and after treatment.

	CCNMES group Mean (SD)	NMES group Mean (SD)	*Z*	*p* value	Difference between groups (95% CI)
aEMG (*μ*V)					
Before treatment	18.69 (16.03)	16.93 (17.35)	−0.440	0.660	1.77 [−8.65–12.18]
After treatment	38.15 (17.55)^a^	28.16 (20.77)^a^	−1.497	0.134	9.99 [−2.02–21.99]
Difference	19.45 (11.30)	11.24 (9.31)	−2.478	0.013	8.22 [1.76–14.67]^b^
iEMG (*μ*V·s)					
Before treatment	43.04 (41.75)	35.29 (36.36)	−0.667	0.505	7.60 [−16.66–32.18]
After treatment	82.91 (43.39)^a^	58.22 (42.04)^a^	−1.824	0.068	24.69 [−1.96–51.33]
Difference	39.86 (24.68)	22.94 (17.79)	−2.402	0.016	16.93 [3.51–30.34]^b^
RMS (*μ*V)					
Before treatment	54.54 (52.03)	44.35 (45.66)	−0.717	0.473	10.19[−20.34−40.71]
After treatment	104.40 (53.58)^a^	72.53 (50.80)^a^	−2.025	0.043	31.87 [−0.70–64.43]
Difference	49.86 (30.76)	28.18 (22.71)	−2.453	0.014	21.68 [4.82–38.54]^b^

SD: standard deviation; CI: confidence interval; aEMG: average electromyography; iEMG: integrated electromyography; RMS: root mean square; ^a^*p* < 0.001, differences before and after treatment; ^b^*p* < 0.05, differences between groups.

## Data Availability

The datasets generated and analyzed during the current study are available from the corresponding author on reasonable request.
